# How Acceptable are Antiretrovirals for the Prevention of Sexually Transmitted HIV?: A Review of Research on the Acceptability of Oral Pre-exposure Prophylaxis and Treatment as Prevention

**DOI:** 10.1007/s10461-013-0560-7

**Published:** 2013-07-30

**Authors:** Ingrid Young, Lisa McDaid

**Affiliations:** MRC/CSO Social and Public Health Sciences Unit, University of Glasgow, 4 Lilybank Gardens, Glasgow, G12 8RZ UK

**Keywords:** Pre-exposure prophylaxis, Treatment as prevention, Acceptability, HIV prevention

## Abstract

Recent research has demonstrated how antiretrovirals (ARVs) could be effective in the prevention of sexually transmitted HIV. We review research on the acceptability of oral pre-exposure prophylaxis (PrEP) and treatment as prevention (TasP) for HIV prevention amongst potential users. We consider with whom, where and in what context this research has been conducted, how acceptability has been approached, and what research gaps remain. Findings from 33 studies show a lack of TasP research, PrEP studies which have focused largely on men who have sex with men (MSM) in a US context, and varied measures of acceptability. In order to identify when, where and for whom PrEP and TasP would be most appropriate and effective, research is needed in five areas: acceptability of TasP to people living with HIV; motivation for PrEP use and adherence; current perceptions and management of risk; the impact of broader social and structural factors; and consistent definition and operationalisation of acceptability which moves beyond adherence.

## Resumen

Investigaciones recientes han demostrado la forma en que los antirretrovirales pueden ser efectivos en la prevención del VIH. En este estudio se revisan trabajos sobre la aceptación de la profilaxis pre-exposición (PrEP, por sus siglas en inglés) y del tratamiento como prevención (TasP) para la prevención del VIH entre los potenciales usuarios. También se consideran con quién, dónde y en qué contexto esos trabajos de investigación han sido llevados a cabo, cómo se ha medido el nivel de aceptación y cuáles brechas de investigación permanecen abiertas. Los resultados de los 33 estudios considerados evidencian una falta de investigación en el campo de TasP y PrEP. En el caso de estos últimos, los estudios existentes se han enfocado en hombres que tienen sexo con hombres en el contexto de los EEUU. Estos resultados también evidencian una falta de investigación en cuanto a las diferentes medidas de aceptación. Con el objetivo de identificar cuándo, dónde y para quién PrEP y TasP serían más apropiados y efectivos, se requieren estudios adicionales en cinco áreas: la exploración de la aceptabilidad de TasP para personas que viven con VIH; desde el punto de vista motivacional sobre el uso y adherencia a PrEP;; acerca de la forma actual en la que se percibe y se maneja el riesgo; el estudio de factores sociales y estructurales más amplios; y por último sobre la consistencia en la definición y sobre el fomento de la aceptabilidad más allá de la adherencia.

## Introduction

The 30 years of the HIV epidemic have been marked by significant successes and failures in biomedical and behavioural research. While a cure or vaccine remain elusive, there have been significant advances in the clinical treatment of HIV [1], the prevention of mother to child transmission [2], and, to a lesser extent, in post-exposure prophylaxis (PEP) [3]. There have also been important community-based advocacy efforts and responses to HIV, including safer sex and risk reduction strategies, which form the basis of behavioural interventions as well as provide better understandings of the social contexts of the epidemic [4, 5]. However, the response to recent clinical trial results such as HPTN 052, Pre-exposure Prophylaxis Initiative (iPrEx) trial, and Partners in Prevention, which use antiretrovirals (ARVS) for prevention, has been significant. Hailed as ‘the beginning of the end of AIDS’ [6], these biomedical developments in HIV prevention provide exciting, if challenging, opportunities for HIV prevention.

Pre-exposure prophylaxis (PrEP) is the use of ARVs by HIV-negative individuals before potential exposure to HIV to prevent transmission. The iPrEx trial examined the efficacy of oral PrEP amongst men who have sex with men (MSM) and transgender women at high-risk of HIV, including HIV negative sexual partners in sero-discordant relationships. A sub-study of the iPrEx trial found that a daily dose of oral PrEP could reduce the risk of HIV transmission by up to 99 % if adherence was high (taking seven tablets or more per week) [7]. In the United States (US), findings from iPrEx and other PrEP trials [8] resulted in the US Food and Drug Administration (FDA) approval of Truvada (emtricitabine/tenofovir disoproxil fumarate) for use as PrEP in July 2012 [9]. This was coupled with the release of Center for Disease Control and Prevention (CDC) interim guidelines on PrEP use for both MSM in January 2011 [10] and heterosexual men and women in serodiscordant relationships in August 2012 [11]. The guidelines advise initial screening for HIV and STIs, ongoing adherence and condom counselling, and 2–3 monthly follow up that includes regular screening for HIV and STIs. However, there have been mixed results from major PrEP trials; studies such as FEM-PrEP and VOICE recently failed to prove the effectiveness of PrEP as a result of poor participant adherence to the drug regime [12]. The US currently remains the only country where ARVs are licensed for use as PrEP. While some countries such as Latvia [13] have reported using PrEP, and HIV clinicians in South Africa have issued guidelines for off-label use for MSM, official implementation has been met with some caution. For instance, the British HIV Association (BHIVA) and British Association for Sexual Health and HIV (BASHH) issued a position statement calling for further research to confirm whether PrEP should be used as an effective HIV prevention option in the UK [14] and the European Medicines Agency (EMA) recently held a consultation on the clinical and non-clinical developments of PrEP in Europe [15].

Treatment as Prevention (TasP), already used effectively to prevent mother to child transmission [2], is also being advocated as a means to manage and reduce sexual transmission of HIV. The HPTN 052 trial found that starting HIV-positive sexual partners on ARVs early (i.e. before their CD4 count dropped below the levels of 200–350 cells/μL advised in international guidelines for treatment initiation at the time of the trial) resulted in a 96 % reduction in HIV transmission amongst heterosexual serodiscordant couples [16]. The results from HPTN 052 have led to changes in treatment guidelines in the US and a number of European countries such as the UK, France and Germany [13, 17, 18]. More recently, the World Health Organisation has called for HIV treatment to be started at 500 cells/μL [19]. These changes highlight how TasP is rapidly establishing itself as a clinically sanctioned method of HIV prevention [20]. However, although hailed as ‘proof of concept’ in relation to ARVs preventing sexual transmission, how TasP should be implemented on population-wide level is less clear and has raised a number of practical and ethical issues [21]. The effectiveness of TasP will rely on the early diagnosis and retention in care of HIV positive individuals, who often experience multiple barriers to accessing services and treatment [22]. This approach also raises the question of whether individuals living with HIV should be asked to start treatment in order to prevent transmission to others, rather than in relation to their clinical needs [23]. These issues need to be addressed before TasP is rolled out at a population level.

Recent findings from PrEP and TasP trials are situated in a longer history of how ARVs are part of and affect HIV prevention. The introduction of highly active antiretroviral therapy (HAART) in the mid-1990s raised concerns that optimism as a result of new HIV treatment might result in an increase in sexual risk behaviour. Research into HIV treatment optimism did not find a consistent association between HIV optimism and high-risk sexual behaviour [24, 25]. More recently, a review of treatment-related optimistic beliefs and risk of HIV transmission found mixed results. While quantitative studies demonstrated a link between optimism and risk of transmission, the review found that inconsistent measures and qualitative findings raise questions about this association and calls for a more comprehensive examination of HIV treatment optimism issues [26]. In 2008, the ‘Swiss Statement’ further complicated HIV prevention messages by stating that people living with HIV who are on treatment, have an viral load that has been undetectable for at least 6 months and do not have an STI are not sexually infectious [27]. Research about issues raised by the Swiss Statement revealed that sexual decision making in relation to HIV prevention was not based solely on calculations of risk, but also on socioeconomic factors [28] and emotional and relationship priorities [29]. These findings suggest that the way in which people understand biomedical prevention methods and incorporate them into the context of their everyday lives will be critical to of the management of HIV-related risks.

There is considerable debate about the ways in which the PrEP and TasP should (and will) be introduced as HIV prevention options. Nguyen et al. [23] have expressed anxieties that ARV prevention will replace behavioural interventions (and the necessary funding of the latter); Padian et al. [1] have called for combination prevention, recognizing the continued need to manage and support behaviour change strategies [30]; and the VOICE trial findings have resulted in calls for improved understandings of what social, cultural and other factors affect adherence [31]. Indeed, rather than separating biomedical from behavioural interventions, a number of authors have characterised PrEP and TasP as bio-behavioural, pointing to how individuals will have to modify their behaviour to take the medication [32, 33]. As in the case of HIV treatment optimism, responses to the Swiss Statement, and the non-adherence to PrEP by participants in the VOICE trial, people’s actual use (or non-use) of PrEP or TasP will have a significant impact on their effectiveness in preventing the sexual transmission of HIV. We need, therefore, to better understand if, how and in what contexts, PrEP and TasP will be acceptable to people seeking to prevent the sexual transmission of HIV.

While PrEP and TasP may reflect different programmatic approaches to the prevention of sexual transmission of HIV—an individual approach to PrEP in contrast to a potential population-wide approach for TasP—there is considerable overlap in how each intervention may be managed at an individual level and how they may or may not be acceptable to trial participants, potential end users and/or their sexual partners. Research on the acceptability of contraception, perhaps the closest comparator for biomedical-based interventions in sexual health, considers acceptability to be the voluntary, sustained use of a product in the context of alternatives [34]. With regards to oral and microbicide-based PrEP in clinical trials, Mensch and colleagues point out that there is little agreement in the literature on how acceptability is defined and measured. They suggest that acceptability should be judged in relation to adherence to the medication, and consider elements such as product attributes, dosing regimen, deliver mechanisms, but also partner’s attitudes and the effects of the product on sexual encounters [35]. Similarly, Golub et al. [33] describe the need to support individuals in their uptake, use and risk-reduction strategies, encouraging them to make rational choices about their sexual health. In this review, we assess the literature on the acceptability of oral PrEP and TasP for the prevention of sexually transmitted HIV amongst potential users. We consider with whom, where and in what context this research has been conducted, how acceptability has been approached, and what research gaps remain.

## Methods

Five major databases (Medline, Psychinfo, Web of Science, Embase and Global Health) were initially searched using the terms in Box 1. Subsequent searches were conducted on PubMed to update the initial search. Articles were included if they:

- examined acceptability of oral PrEP and/or TasP as HIV prevention options in the context of sexual transmission;
- described primary data collection with current or potential oral PrEP or TasP users (including empirical studies, embedded studies in larger RCTs and process evaluations);
- were published in English; and
- were published between January 2008 (in order to capture research related to recent ARV developments) up to December 2012.

**Box 1:** Search terms used

MESH terms used	Free text search terms used
‘HIV infections’ ‘HIV infections/prevention & control*’ ‘Antiretroviral therapy, highly active’ ‘Patient acceptance of health care’	HIV ‘Human immunodeficiency virus’ ‘human-immunodeficiency-virus’ HIV prevention TAP TASP ‘Treatment as prevention’ ‘treatment-as-prevention’ PREP ‘pre-exposure prophylaxis’ ‘pre exposure prophylaxis’ ‘preexposure prophylaxis’ ‘Highly active antiretroviral treatment’ HAART ‘treatment outcome’ feasib* acceptab* adoption attitude*

The earliest study identified in our search on the use of oral ARV-based technologies for the prevention of sexually transmitted HIV was published in 2008 and reflects the start-point for this review. Editorials or comment pieces were excluded as they did not include primary data collection. Of a total of 90 articles found, 33 met the inclusion criteria. These articles were all sourced from peer-reviewed journals. A number of conference abstracts were identified, but were excluded due to insufficient information about how acceptability was measured and limited information on findings. Formal quality appraisal of the publications was not undertaken as the aim of this review was to establish a broad overview of research available, including how acceptability was assessed, where studies have been conducted, and what the key findings were in relation to acceptability. Assessment was made of individual articles, even when multiple articles were from the same study. The first author identified key characteristics of each study in accordance with STROBE (Strengthening the Reporting of Observational studies in Epidemiology) checklist [36]. This specifically included: participants; setting; type of intervention; study aims; study design and methods; study size; measures of acceptability; key results; and interpretations. The second author reviewed the identification and organisation of key characteristics and verified these findings.

## Results

Of the 33 articles and reports in our review, 30 addressed the acceptability of PrEP, reflecting 27 separate studies, and three addressed the acceptability of TasP. A detailed overview of each publication is included in Table 1.

**Table 1:** Overview of pre-exposure prophylaxis (PrEP) and treatment as prevention (TasP) acceptability studies

Study	Participants	Setting	Type of intervention	Study aims	Study design and methods	Study size	Measures of acceptability	Key results	Interpretations
Aghaizu et al. [55]	Men who have sex with men (MSM)	London, UK	Daily PrEP	Assess current and intended future use of PrEP	Cross-sectional survey Self-administered survey	842	Asked: if likely to take a daily dose (oral) of PrEP if available; used 5 point Lickert scale	Half reported likely to use PrEP; associated with <35 years, UAI with casual partners and previous use of PEP	PrEP in form of daily pill acceptable to half of sexually active MSM in London
Amico et al. [70]	Heterosexual and MSM Sero-discordant couples (SDCs)	Brazil, Ecuador, Peru, South Africa, Thailand, and the US	Daily oral PrEP	Refine/improve adherence reporting and support mechanisms in iPrEX study sites	RCT-embedded (iPrEX) Needs assessment and intervention development with trial participants and staff	n/a	n/a	Social stigma affects adherence reporting	Suggests separation of reporting and counselling could improve reporting and adherence
Barash and Golden [45]	MSM	Seattle, US	Daily oral PrEP	Explore men’s willingness to use PrEP	Cross-sectional survey Self and interview administered questionnaires at Gay Pride event and STI Clinic	215	Asked: if taking HIV medicines helped prevent HIV, would you take them everyday*?*; yes/no question	44 % were willing to take daily PrEP; no association between risk behaviour and interest in PrEP; low income, <40 years and recruitment from STI clinic associated with interest in PrEP	MSM are aware of PrEP and interested in the intervention
Brooks et al. [46]	MSM SDCs	Los Angeles, US	Daily oral PrEP	Identify factors that may facilitate or impede future adoption of PrEP amongst MSM in serodiscordant relationships	Cross-sectional study Semi-structured interviews with MSM recruited from community settings	50	Participants were informed PrEP was 90 % effective and asked a range of questions around: opinions of PrEP; reasons why they might/might not want to use PrEP; if they had concerns with taking PrEP; perceptions of transmission risk; feelings about safer sex while taking PrEP; information desired about PrEP; feelings about non-infected partner taking PrEP	Motivators for adoption included protection against HIV infection, the opportunity to engage in unprotected sex. Concerns and barriers to adoption included the cost of PrEP, short- and long-term side effects, adverse effects of intermittent use or discontinuing PrEP, and limited accessibility	Need for carefully planned and implemented PrEP delivery programme which includes education and counselling interventions
Brooks et al. [47]	Se MSM SDCs	Los Angeles, US	Daily oral PrEP	Examine risk behaviour, acceptability and potential adoption of PrEP	Cross-sectional study Mixed methods: semi-structured interview and survey with 25 couples recruited from community settings	50	PrEP described as 90 % effective. Survey questions had participants agree/disagree with range of statements relating to: PrEP for HIV prevention; risk compensation/disinhibition; HIV stigma; PrEP adoption intentions in relation to PrEP use by others	64 % of participants indicated a likely increase in sexual risk behaviour, and 60 % indicated an abandonment of condom use when using PrEP	PrEP should only be offered as part of a comprehensive HIV prevention strategy which includes risk reduction counselling to help moderate risk compensation
Dombrowski et al. [67]	HIV-positive patients	Seattle, US	TasP	Evaluate the acceptability of including prevention considerations in decision-making about ART initiation and to gauge the population’s interest in starting ART for prevention	Cross-sectional survey Anonymous, self-administered questionnaire in STI clinic	136	Participants were asked: if they would take HIV medicines in order to make it less likely that they would pass the virus on to others; if doctors should offer HIV medicine to patients to decrease transmission; if being on treatment means PLHA are less likely to pass the virus on in unprotected sexual encounters	56 % expressed interest in starting ART specifically to decrease the risk of transmitting HIV to sexual partners; majority believed doctors should offer patients ART for this purpose (61 %); 35 % reported having UAVI with partner of negative or unknown status in preceding year; respondents interested in early start ART were more likely to report non concordant UAVI; >50 years associated with lower interest in early start; knowledge of ART as prevention was not widespread	Public health efforts should be designed to ensure that patients have an opportunity to consider early initiation of ART, including the potential prevention benefits of treatment as this would be acceptable to many PLHA
Eisingerich et al. [64]	Female sex workers (FSW), MSM, intravenous drug users (IDU), serodiscordant couples (SDC)	Peru, Ukraine, Kenya, Uganda, Botswana, South African and India	Daily oral PrEP; Intermittent PrEP; Injectable PrEP	Explore attitudes, preferences towards hypothetical and known attributes of PrEP programs and medication (oral/parenteral) and future acceptability	Cross-sectional survey Interview and self-administered questionnaires	1,790	Asked questions using 4-point Lickert scale around: adherence to other medications; willingness to take PrEP and associated key feelings; potential barriers to use. Conjoint analysis used to assess preferred: PrEP route; access; time spent accessing PrEP; frequency of testing	61 % reported a willingness to use PrEP, even if they have to pay for it and in spite of potential side effects, cost and frequent HIV testing	Willingness among key populations to use PrEP if it was efficacious and affordable; long lasting injection would be a good target for PrEP
Galea et al. [63]	FSW, Male–Female transgendered persons (TG) and MSM	Lima, Peru	Daily oral PrEP; Intermittent PrEP	Explore social issues associated with PrEP acceptability and preference for PrEP delivery	Cross-sectional qualitative study Focus groups and conjoint analysis with 8 hypothetical PrEP scenarios	45	Focus group questions asked about: PrEP knowledge and attitudes to daily use; reasons for/against willingness to take PrEP; social and health provider concerns with PrEP use; concerns with PrEP access, cost, side effects, etc.; potential behavioural change. Presented range of efficacy 75–95 %. Applied conjoint analysis to assess preferences for 7 PrEP characteristics	Little awareness of PrEP; general acceptance of PrEP as an option, most affected by cost and side-effects; TG and MSM described potential decrease in condom use if PrEP available; stigma and discrimination associated with PrEP use, along with and mistrust of health-care professionals	Acceptability of PrEP as HIV prevention method; need to consider the distinct needs and concerns of FSW versus TG and MSM through deeper exploration
Galindo et al. [53]	MSM, Male–female TG	California	Daily oral PrEP	Examined acceptability of PrEP and barriers to community uptake amongst communities most affected by HIV in the US	Cross-sectional qualitative study Semi-structured interviews	30	Authors reported on willingness to take daily ARV and concerns about: following medical monitoring; side effects; long-term health risks; associated cost and financial considerations; reasons for starting/stopping PrEP; and perceived effects of PrEP in the community	General interest expressed in PrEP. Barriers include: lack of community awareness of and confusion about PrEP; concerns about PrEP use; concerns about how a PrEP intervention could be packaged and marketed appropriately	Authors suggest: need to consider importance of how PrEP information framed and accurately delivered; recommend community mobilization strategies in PrEP delivery; PrEP delivery can help address mistrust of medical setting if combined with sociobehavioural approaches; need to consider if PrEP is actually cost-effective and equitable option for diverse populations
Golub et al. [33]	HIV-negative MSM	New York, US	Daily oral PrEP	Explore awareness of and attitudes toward PrEP in high-risk population; aims to understand the psycho-social and behavioural factors which enhance/reduce motivation for risk reduction in relation to PrEP	Cross-sectional survey Audio computer assisted self-interview administer survey; with semi-structured interview to assess substance use and sexual risk	180	Instructed to presume PrEP 80 % effective; using 5-point Lickert scale, asked participants: if they would take PrEP; how taking PrEP would affect condom use	23 % had heard of PrEP; 68.5 % likely to use PrEP with no association in age, income or education; 35 % said they would decrease condom use	PrEP could be used by MSM already engaging in high risk activity who are unlikely to use condoms; findings support both behavioural disinhibition and risk compensation models; highlights importance of developing behavioural interventions to accompany any wide-scale provision of PREP to high-risk populations
Guest et al. [38]	Heterosexual women, 18–35 years	Tema, Ghana	Daily oral PrEP	Assess acceptability of daily PREP	RCT-embedded; part of the TDF West African oral PrEP trial Structured survey and in-depth interview	361 (survey); 24 (interview)	Acceptability (to daily pills) measured through adherence rates	Self-reported adherence—82 %; identified social relationships and travel constraints as barriers to adherence	PrEP acceptability high if efficacious and few side effects; need to consider how social and structural factors will affect adherence
Guest et al. [37]	Heterosexual women, 18–35 years	Tema, Ghana	Daily oral PrEP	Explore risk disinhibition and compensation; maps changes in sexual risk behaviour	RCT-embedded; part of the TDF West African oral PrEP trial Structured survey and in-depth interviews	361 (survey); 24 (interview)	Acceptability measured through daily adherence rates and completion of 12 follow up visits	Number of sexual partners and rate of unprotected sex acts decreased across the 12-month, with some exceptions; adherence support through counselling deemed effective	HIV prevention does not lead to risk disinhibition; counselling can be effective in supporting adherence and risk behaviour; need to consider how structural factors cannot be addressed through individual interventions
Holt et al. [58]	MSM	Australia	Daily oral PrEP; Intermittent oral PrEP	Investigate the willingness of PrEP use and likelihood of decreased condoms use	Cross-sectional survey Online survey	1,161	Survey asked 25 attitudinal questions relating to PrEP using 5-point Lickert scale; established a 7-item scale assessing willingness to use PrEP, including statements relating willingness to: if PrEP was considered, when PrEP was taken, effectiveness of HIV prevention, and payment for PrEP	28 % of participants willing to use PrEP, associated with younger age, having anal intercourse with casual partners; 8 % of men who were willing to use PrEP were less likely to use condoms; decreased condom use independently associated with older age, UAI with casual partners and perception of increased risk of HIV	MSM cautiously optimistic about PrEP; minority of men who expressed willingness appear to be appropriate candidates given they engage in UAI and perceive themselves at risk of HIV
Holt et al. [69]	MSM	Australia	TasP	Assessed attitudes towards medicines, HIV treatment and ARV-based prevention amongst HIV-positive and HIV-negative MSM	Cross-sectional survey Online survey	1041 (919 HIV-neg; 122 HIV-pos)	Survey asked about attitudes to treatment and treatment as prevention, including beliefs about: role of viral load in transmission; role of being on treatment in transmission; concerns with side-effects; ease of taking medication daily	HIV-neg and HIV-pos MSM have similar views of PrEP as effective; MSM sceptical that treatment for HIV-pos men can reduce onward transmission risks, especially HIV-neg men	MSM in general are supportive of PrEP being made available; MSM more sceptical of ARV-based prevention for HIV-positive MSM; suggests a bias towards prevention tools for HIV-negative MSM
Hosek et al. [43]	MSM, 18–22	Chicago	Oral PrEP	Examined acceptability and feasibility of PrEP and PrEP study	Behavioural and biomedical HIV intervention trial. Questionnaire (study-design); time-line follow-back interview (adherence)	58	Adherence was measure of PrEP acceptability within the trial; HIV risk behaviour/compensation assesses with 6-item scale	Acceptability of study design was high; lower acceptability of drug and regimen; higher acceptability of PrEP with non-pill group;	PrEP use amongst young MSM will require behavioural interventions to maintain/support adherence; future studies should begin on the effectiveness of PrEP amongst adolescents under 18 at risk of HIV
Jackson et al. [61]	MSM	China	Oral PrEP	Identify predictors of lower vs higher willingness to use PrEP to reduce HIV amongst MSM	Cross-sectional survey Self-report questionnaire, recruited through community outreach organisations, locations and events	570	Asked range of questions relating to: beliefs about HIV severity; personal HIV risk; PrEP related stigma, benefits and self-efficacy in taking PrEP	Degree of willingness to use PrEP was related to previous consultation about HIV, barriers to condom use, increased depressive symptoms; belief that intervention was low in stigma and was high in potential benefits	Highlights the importance of intervention-specific beliefs as well as how psychological status, sexual behaviour and demographic factors influence willingness to adopt prevention strategies
Kalichman et al. [68]	Men, women and transgendered people living with HIV (PLHA)	Atlanta, Georgia	TasP	Explore sexual behaviour amongst HIV + people recently diagnosed with an STI; explore knowledge of viral load and treatment optimism	Cross-sectional survey Audio-computer assisted structured interviews in AIDS service organisations, health care providers, social service agencies and infectious disease clinics	490	4 questions, using 6-point Lickert scale, asked about: levels of infectiousness; role of treatment in prevention; feelings around treatment and condom use	14 % of PLHA in study diagnosed with STI in previous 6 months; individuals with new STI had more sexual partners, including serodiscordant partners; participants with STIs more likely to have detectable viral loads and less likely to know their viral loads than participants who did not contract an STI; believing in an undetectable viral load leading to lower infectiousness was associated with a new STI	Need to integrate STI diagnostics and treatment into routine clinical services; patients should be taught how to recognize early STI symptoms; scale-up of TasP will need to address infectiousness beliefs and rapid detection and treatment of STIs
Krakower et al. [49]	MSM	Boston, US	Daily oral PrEP	Aims to assess knowledge of PrEP amongst MSM in light of iPrEX study findings	Cross-sectional survey Cross-sectional internet-based survey through MSM social networking site before and after publication of iPrEX results were published	398 pre-iPrEX; 4558 post-iPrEX	Participants were asked questions relating to PrEP knowledge, interest and experience in PrEP use, adapted from Mimiaga et al. [51] (See below)	Limited awareness of PrEP before (11 %) and after (19 %) publications; interest in PrEP high before (76 %) and after (79 %); awareness of PrEP associated with awareness of PEP; interest in PrEP use associated with older age, UAI, more than one partner in 3 months and perceiving oneself to be at increased risk of HIV	Need for information campaigns to raise PrEP awareness and facilitate dialogue amongst MSM and their health providers; need to learn more about barriers to discussing HIV risk behaviours and starting PrEP in a clinical setting
Leonardi et al. [56]	Men (mostly MSM)	Toronto, Canada	Oral PrEP	Assess current levels of awareness and usage of PrEP amongst ‘high risk’ men	Cross-sectional survey Self-administered questionnaire by men undergoing rapid HIV test in sexual health clinic	256 (195MSM; 61 non-MSM)	Survey questions asked about willingness to use PrEP using a 4-point Lickert scale	11.7 % were aware of PrEP (more MSM than non MSM); willingness to consider PrEP amongst MSM was 66 % and 49 % amongst non-MSM; willingness was associated with high-risk activities	PrEP appears to be acceptable to high-risk MSM; need further, multistakeholder research on how to best roll out PrEP
Lorente et al. [57]	MSM	France	Intermittent oral PrEP	Assess interest in participating in an ‘on-demand’ PrEP trial and identify individual and structural barriers	Cross-sectional survey Self-administered questionnaires in gay venues and online	527	Questions asked relating to future study participation and potential PrEP use based on 4-point Lickert scale; questions focused on willingness to take PrEP, clinical follow up preferences, impact on condom use, and problems with taking medication	40 % said they would be interested in participating in a PrEP trial; of these men, most concentrated sexual activity 2–3 days a week; only 19 % of these men said they would not know that they would have sex 24 h before the event; lower education levels and higher-risk sexual behaviour with multiple partners associated with willingness	Significant acceptability for participation in ‘on-demand’ PrEP trial; encouraging for research aimed at testing additional HIV prevention methods which are potentially less toxic, less expensive and improved adherence than daily PrEP
Mansergh et al. [50]	MSM	Chicago, Los Angeles, New York and San Francisco, US	Oral PrEP	Examine use and sharing of ARVs for the purposes of PrEP and PEP among high-risk substance using MSM	Behaviour Intervention Trial (Project MIX) Baseline and 12-month follow-up assessments	1,011	n/a	PEP and PrEP being used by 2-4 % of HIV-negative MSM or given to sexual partners by a similar percentage of HIV-positive men to prevent infection; optimism about HIV treatment was predictive of later PrEP and PEP use for HIV-negative men	Research will be needed to determine the best behavioural messages and counselling approaches that combine condom use with PrEP
Mimiaga et al. [51]	HIV-negative MSM	New England	Oral PrEP	Assess prior PrEP use and awareness and future intent to use PrEP	Cross-sectional survey Interview-administered survey	227	Participants were asked a range of questions relating to awareness and willingness to use PrEP, including awareness and use of PrEP. Participants were also presented with specific hypothetical scenarios to identify under which conditions they would be more likely to use PrEP	19 % heard of PrEP; knowledge of PrEP was associated with higher income; 74 % intended to use PrEP if available once made aware of what it was; willingness to use PrEP was associated with lower levels of education; high numbers of men willing to take PrEP daily, or intermittently and for all UAI	PrEP would be acceptable to MSM in this study; findings suggest need for tailored educational messages to ensure appropriate PrEP if rolled-out
Minnis et al. [40]	HIV-negative women	South Africa, Uganda and United States	Daily oral PrEP and Topical PrEP	Compared short-term adherence to and acceptability of three PrEP regimens	Acceptability Trial Interviews: face-to-face at 6 weeks into trial; in-depth interviews with 25 % participants	168; 36 in-depth interviews	Self reported adherence on 5-point Lickert scale; future willingness asked using 4-point Lickert scale; from interviews, authors reported on: product characteristics, partner relations, stigma associated with taking tablets and sexual pleasure	93 % reported future use of daily tablet; 83 % likely to use gel; 82 % likely to use dual products; preference for mode of delivery varied according to location; participants identified HIV and illness stigma, product characteristics, male partners as barriers	Need to consider geographic and cultural experience with formulation, male partner involvement and broader sexual health benefits
Mutua et al. [41]	MSM and FSW	Kenya	Daily and intermittent oral PrEP	Compare safety and drug adherence between daily and intermittent PrEP	Clinical trial Followed for 4 months; sexual activity data collected via daily text and interview	67 MSM; 5 FSW	Main outcome measures included: willingness to use study regimen if effective; adherence rates for daily and intermittent dosing	83 % adherence for daily PrEP, 55 % for intermittent and 26 % for post-coital; change in regular routine, alcohol use, social stigma and travel identified as barriers to adherence	Adherence to intermittent dosing regimens, fixed doses and in particular coitally-dependent doses may be more difficult than adherence to daily dosing
Nodin et al. [52]	MSM	New York, US	Oral PrEP, microbicides	Explore knowledge and acceptability of four biomedical products/technologies, as alternatives to condoms	Cross-sectional qualitative study Semi-structured interviews	72	Authors report on knowledge of and response to PrEP; issues raised include: ease of use, use as an alternative prevention strategy, concern with side effects and efficacy, and concerns with behavioural impact	Almost no knowledge of PrEP; limited knowledge of microbicides and some information about PEP; reaction to all products were positive and men were willing to take part in research, with the exception of PrEP; responses to PrEP were polarized as either enthusiastic of negative; negative reactions focused on presumed efficacy and side-effects; concern that these products would encourage barebacking	PrEP will require the most work of all prevention methods explored in this study to improve acceptability amongst MSM who engage in UAI so as not to increase risk factors
Poynten et al. [59]	HIV-negative MSM	Sydney	Rectal microbicides and PrEP	Explore the awareness of rectal microbicides and willingness to participate in rectal microbicides and PrEP trials	Cross-sectional study Structured face to face interviews; quantitative sexual behaviour data on unprotected insertive and receptive anal sexual collected	1,427	Participants were asked two questions about PrEP, using 5-point Lickert scale, including: if they had used ARVs for prevention; if they would participate in a trial	43 % men willing to participate in PrEP drugs trials; higher among those who reported unprotected anal intercourse with HIV-positive partners; no evidence of current PrEP use	Extensive community around PrEP and any potential role it might play in HIV prevention, would be required before PREP could be trialled amongst MSM
Saberi et al. [54]	MSM SDCs	San Francisco	PrEP	Explore PrEP awareness, concerns relating to PrEP provision and establish uptake by HIV-negative partner	Cross-sectional study Mixed methods: Audio computer Assisted self Interview survey and semi-structured face to face interviews	164 couples (328 men); 16 SDCs interviewed (32 men)	Survey questions asked: how likely would it be for you/your partner to take PrEP; should PrEP be offered to anyone who wants to take it. Qualitative interviews explored: knowledge of PrEP; source of knowledge; experience of taking PrEP; PrEP discussions with partner; if PrEP should be offered and potential issues	62 % heard of PrEP (1/4 mistook PEP for PrEP); low endorsement of PrEP uptake and 40 % uncertain if PrEP should be offered; uptake of PrEP by couples associated with insertive UAI and negatively associated with receptive UAI	Suggests need for further PrEP education within communities; need to determine appropriate groups within which PrEP can have the highest impact
Smith et al. [66]	African–American heterosexual men and women, 18–24; African–American MSM	Atlanta, Georgia	Daily oral PrEp	Explored attitudes and service access preference for daily PrEP	Cross-sectional qualitative study Focus groups	8 FG with heterosexual men and women (*n* = 58); 2 FG with MSM (*n* = 19)	Authors reported on concerns regarding: rapid HIV testing; dispensing location and cost; side effects and burden of taking daily medication; effectiveness; risk perception; social response of peers; risk compensation	Participants reported significant interest in PrEP. Interest was associated with low cost, PrEP effectiveness and ease of accessing services and medication; regular, oral testing was not seen as a barrier to PrEP use	Education and counselling efforts to accompany PrEP will need to address: sharing or borrowing of ARVs for PrEP use; reduced adherence; and providing accurate information about the efficacy and necessary duration for PrEP use
Van der Elst et al. [42]	MSM and FSW	Kenya	Daily and Intermittent oral PrEP	Explore experiences of using PrEP for HIV prevention within a clinical trial	Clinical trial Focus groups and semi-structured interviews	10 FG (*n* = 44); interviews (*n* = 7)	Authors reported on: likes and dislikes of pill; dosing schedules; adherence measures; experience with procedures	High acceptability of PrEP, but change to product characteristics would improve acceptability; concerns around the social consequences of PrEP, especially in relation to stigma; alcohol-use, transactional sex and travel challenges to daily and intermittent adherence	Identified wide range of creative and commonplace strategies to help adherence; behavioural adherence interventions will be very important for PrEP success; importance of providing information about partial efficacy in a clear way; PrEP needs to be accompanied by counselling to encourage risk reduction and regular testing
van Griensven et al. [60]	HIV-negative MSM	Bangkok	Intermittent PrEP	Examine frequency and planning of sex.	Cohort study Audio-computer assisted self-interview	823	n/a	86 % reported having sex on 2 days per week or less, and 65 % reported their last sex to have been planned; being aged 22–29 years, not identifying as homosexual, having receptive anal intercourse, and not engaging in group sex were associated with unplanned sex	Most men were exposed to HIV infection only intermittently, and had a window of opportunity to take PrEP prior to sexual activity; suggests intermittent PrEP more appropriate than daily regimen; those lacking opportunity to take intermittent PrEP were at high behavioural risk
Ware et al. [39]	Heterosexual serodiscordant partners	Uganda	Daily PrEP	Explore PrEP adherence in stable HIV serodiscordant heterosexual couples, including barriers and facilitators to adherence	Part of Partners in PrEP RCT Qualitative Interviews	60 (45 participants; 15 partners)	Authors reported on: experiences of taking study pills; descriptions of specific missed doses; descriptions of longer adherence lapses; impact of serodiscordance on the relationship	Adherence motivated by doing no harm to negative partner; relied on family and counselling support, as well as creative daily reminders; adherence lapses linked to breakdown of relationship; adherence links to cultural factors in relation to marriage, children and economic circumstances	PrEP adherence for SDCs may be linked to couples’ determination to resolve the discordance dilemma; use of PrEP in the context of partnered relationships may be associated with improved adherence and effectiveness
Whiteside et al. [65]	Men and women (mostly heterosexual, African American)	South Carolina	Oral PrEP	Determine risk perception and attitudes about PrEP	Cross-sectional survey Self-administered survey in STI clinic	405	Survey questions asked about: risk factors/sexual history; knowledge of PrEP for HIV infection; attitudes and beliefs regarding the use of PrEP for HIV infection, including self-perceived risk of HIV infection and use of PrEP in conjunction with condoms	Most men did not perceive themselves to be at risk of HIV infection; MSM respondents more likely to have heard of PrEP; attitudes towards PrEP affected by gender, age and number of sexual partners in previous 3 months	Awareness and acceptability of PrEP significantly affected by gender, age and number of sexual partners; need to address the gap between self-perceived and actual levels of risk
Zhou et al. [62]	MSM	Beijing, China	Daily PrEP	Investigate awareness and acceptability of PrEP	Cross-sectional survey Interview administered survey	152	Survey asked about willingness to use PrEP using a 4-point Lickert scale. Supplementary questions asked about: side effects, efficacy, HIV stigma, cost and sexual exclusion	11.2 % had heard of PrEP; 67.8 % were willing to use PrEP. Willingness to use PrEP was associated with: inconsistent condom use; never heard of ARV side effects. 35 % of those willing to try PrEP reported a likely decrease in condom use	Sexual behaviour characteristics and knowledge about ARVs may affect willingness to accept PrEP

### PrEP Studies

Eight articles were based on non-clinical research embedded in six separate larger clinical PrEP trials. Three articles, from two trials, focused on heterosexual women’s experiences in serodiscordant relationships in African countries [37–39], with an additional article focusing on heterosexual women’s experiences in South Africa, Uganda and the US [40]. Two articles from one trial in Kenya explored acceptability and willingness amongst MSM [41, 42] while one article focused on MSM in the US [43]. The eighth article described the identification, development and implementation of procedures within the iPrEx study that intended to improve product use and self-report across all the trial sites [44].

Twenty-two PrEP articles were from non-clinical trial research and the majority of these focused on MSM. Ten articles, from nine studies, were with MSM in the US [45–54]. Other articles focusing on MSM included research from the UK [55], Canada [56], France [57], Australia [58, 59], Thailand [60], and China [61, 62]. Galea et al. [63] focused on MSM, female sex workers, and transgendered people in South America (Peru), as did Eisingerich et al. [64], who also included intravenous drug users, serodiscordant couples and young women in Ukraine, India and four countries in Africa. Only two (US) studies explored attitudes towards PrEP amongst heterosexual men and women [65, 66]. The majority of these studies focused on daily oral PrEP, with only five exploring attitudes towards intermittent, or in some cases topical, PrEP use [57, 59, 60, 63, 64] (Table 2).

**Table 2:** Breakdown of PrEP articles by participants and setting

Participants		Setting	No. of articles	No. of studies
Clinical trial PrEP research				
Heterosexual women		Africa	4	3
Men who have sex with men (MSM)		Africa	2	1
MSM		US	1	1
Heterosexual and MSM		Multi-country	1	1
Total			8	6
Non-Clinical trial PrEP research				
MSM^a^		US	10	9
MSM		UK, Canada, France, Australia	5	5
MSM^b^		China, Thailand, Peru	4	4
Serodiscordant Couples (SDC) MSM and Transgender (TG)		Multi-country studies	1	1
Heterosexual men and women^c^		US	2	2
Total			22	21

^a^Galindo et al. [53] also include TG participants

^b^Galea et al. [63] also include TG and female sex worker participants

^c^Smith et al. [66] also include small number of MSM participants

### TasP Studies

Two of the three TasP studies were conducted in the US [67, 68], and the other was Australian [69]. One explored acceptability of TasP amongst people living with HIV but not currently on treatment in Seattle, Washington [67]. A second article examined beliefs around undetectable viral loads and STI acquisition amongst men, women and transgendered people living with HIV in Atlanta, Georgia [68]. A third study explored attitudes towards ARVs, HIV treatment and ARV-based prevention amongst both HIV-positive and HIV-negative participants in Australia [69].

### Measures of Acceptability

The articles used a range of approaches to assess and/or measure acceptability of either PrEP or TasP. PrEP acceptability research embedded within RCTs used individual adherence rates as a measure of acceptability. For those PrEP studies outside of clinical research, the measurement and/or assessment of acceptability varied greatly. Four articles reported on willingness to use PrEP [45, 54–56]. Eight articles reported further on multiple attitudinal issues such as willingness to take PrEP, HIV prevention, access to PrEP, perceptions of personal risk, stigma and continued condom use [48, 49, 51, 57, 58, 61, 62, 64, 65]. Two of these reported on scenarios or conjoint analysis [51, 64]. Qualitative-based articles were less specific about particular questions, but reported a wide range of acceptability issues, including: perceptions of transmission risk; feelings about safer sex; information desired about PrEP; concerns with cost, access, side-effects and regular testing [46, 47, 53, 63]. Measurements of TasP acceptability were similar in variation. Two studies reported on interest in TasP [67, 68]. One study reported on scepticism that reduced viral loads would prevent HIV, as well as concerns about side-effects, ease of taking medication and stigma-related issues [69].

Although two studies [53, 66] highlighted the importance of how information regarding biomedical interventions is framed and delivered to communities, few of the articles reported on how they explained PrEP or TasP efficacy to participants. Five articles presented PrEP efficacy rates to participants of 80 % [48], 90 % [46, 47], or a range of scenarios between 75 and 95 % efficacy [63], and 50 and 75 % efficacy [66]. However, the majority of studies did not specify efficacy of PrEP to participants, or did not report doing so. Similarly, TasP studies did not present precise information about the efficacy of the intervention. Participants in the study by Dombrowski et al. [67] were told that they were less likely to transmit HIV while on medication, but that no one knew for certain. Two more TasP studies did not present participants with information about TasP but asked a range of questions to gauge beliefs about HIV medicines and infectiousness [68, 69].

### Adherence as Acceptability

Three of the PrEP studies in clinical trials reported high levels of adherence to daily pills, ranging from 82 to 93 % [38, 40, 42]. However, Amico et al. [70] noted discrepancies between self-report and actual adherence rates in their needs assessment of adherence reporting in the iPrEx trial. They described the way social stigma played an important role in how adherence was reported; the combination of pill monitoring and adherence counselling did not provide the space for participants to explore their adherence difficulties without feeling judged. In the *Partners in Prevention* trial in Uganda, participants’ adherence was significantly affected by a desire to do no harm to their partner, and by the marital, family and economic circumstances of the positive partner [39].

### Willingness as Acceptability

Studies that were not part of larger PrEP clinical research trials reported limited knowledge of PrEP, ranging from 11 % [56] to 23 % [33]. One study [54] did report 62 % of participants had heard of PrEP, although one-quarter mistook PEP for PrEP. Levels of willingness to use PrEP ranged from 28 % [58] to 80 % [47]; eight out of eleven studies offering numerical findings reported willingness levels of 50 % or more [47–49, 51, 55, 56, 62, 64]. All of the studies reporting willingness in this way focused on MSM. Factors associated with willingness to use PrEP were mixed and there were no strong similarities across the studies. Greater willingness was associated in some studies with lower levels of education [51] and younger age [45, 55]. Four studies reported an association between willingness to take PrEP and unprotected anal intercourse (UAI) [49, 54–56], while Zhou et al. [62] reported an association between PrEP willingness and inconsistent condom use. However, Golub et al. [33] found no association between willingness and age, income or education, and Barash and Golden [45] found no association between willingness and risk behaviour. Holt et al. [58] were unusual in reporting low rates of willingness in their Australian study. Factors associated with willingness in this study were younger age, UAI with casual partners, and increased risk-perceptions. Despite relatively high willingness to take PrEP in most studies, anxieties around medication side-effects were also commonly reported [46, 52, 63, 64]. Only two studies explored associations with PEP experience and none explored knowledge of and/or experience with earlier generations of ARVs [50, 55].

In the TasP studies, knowledge of this prevention method was low. In one, 56 % of HIV positive participants expressed an interest in starting treatment early, while 61 % of participants felt doctors should offer early ART as an option to prevent onward HIV transmission [67]. Interest in starting treatment in this study was associated with being younger than 50. Holt et al. [69] reported that MSM in Australia were sceptical that TasP could reduce onward transmission risks, although HIV-positive participants were less sceptical than HIV-negative participants.

### Risk Compensation and Disinhibition

Much of the research addressed the use of condoms and sexual risk behaviour in combination with PrEP or TasP in some capacity. Where reported, studies embedded in RCTs described ongoing or even increased condom use in combination with PrEP use amongst participants [38]. Outside clinical trials, where PrEP was not yet available, studies could only investigate hypothetical condom use and two with MSM found that significant numbers of participants predicted decreased condom use if PrEP were to be taken [47, 63]. However, other studies with MSM predicted a minimal decrease in condom use [48, 58, 62]. One study described conflicting concerns amongst MSM that PrEP could encourage UAI or could provide an additional safety measure if condoms were not used [52]. One of the TasP studies reported how participants took viral load into account in relation to sexual risk and that having a low viral load was associated with recent STI acquisition [68]. The authors suggested that beliefs around infectiousness and increased risk of STI acquisition need to be addressed.

Few of the studies explored if, or how, participants thought they were at risk. Four studies explicitly asked about self-perceptions of risk [49, 53, 58, 65]. Of these, all but one found most participants did not perceive themselves to be at high risk of HIV [53]. Studies that included participants in serodiscordant relationships did not explicitly explore perceptions of risk. Brooks et al. [46] did explore how serodiscordant MSM couples managed risk and found that most relied on condoms, although strategic positioning and withdrawal were also reported. Golub et al. [48] reported that PrEP could reduce perceptions of HIV risk amongst MSM who would see unprotected sex as an acceptable risk in the context of PrEP (risk compensation), but also reduce the behavioural constraints for men who desire condomless sex (behavioural disinhibition). This distinction between compensation and disinhibition was only explored within a few studies. In addition, both Golub et al. [48] and Holt et al. [58] found that some MSM who reported non-condom use and high risk sexual behaviour also indicated a willingness to use PrEP. The authors concluded that non-condom use for these men may not be risk compensation (the replacement of condoms with PrEP); PrEP use would instead signal an *increase* in response to risk of HIV because condoms were never or rarely used.

### Social and Structural Issues

Social and structural influences on adherence or willingness were only considered by seven studies. Amico et al. [70] demonstrated how social desirability to be an adherent trial participant may play in both the reporting and management of PrEP adherence. Ware et al. [39] described how the social context, especially in relation to intimate and/or marital relationships, was integral to adherence as well as to how HIV risk management is negotiated on a personal, intra-personal and socio-cultural level. Looking to a broader social context, Galindo et al. [53] reported on the importance of awareness and understanding of PrEP within communities and the need to address mistrust of medical settings. Guest et al. [38] found that in addition to individual counselling, structural constraints such as financial concerns, access to health services and HIV-related social stigma needed to be considered when assessing and responding to adherence challenges. Similarly, Galea et al. [63] identified structural barriers to PrEP access and adherence, such as cost of PrEP, mistrust of health providers and discrimination in relation to PrEP use. Where Smith et al. [66] reported on the importance of low cost in relation to willingness, and Galindo et al. [53] reported cost as a significant barrier, Eisingerich et al. [64] found that cost itself was not always a barrier as participants were willing to pay for PrEP.

## Discussion

This review found that acceptability research in relation to PrEP and TasP was highly unbalanced in terms of population, geographic diversity, and research context. Notably, we found only three TasP acceptability studies and PrEP acceptability research which focused primarily on MSM in a US context. We also found varied accounts of acceptability and little reporting or consistency of how interventions were presented to participants. While levels of willingness to use PrEP were high amongst the majority of study participants, little attention was given to the importance of risk perception, as well as to how social and structural influences would impact PrEP or TasP acceptability. Our findings have important implications for future research, intervention implementation and scale-up. First, we will discuss the limitations of this study.

We considered only peer review published studies. Although a number of research gaps have been identified, this is a fast moving field and the review may have missed other studies have not yet been published in peer review journals. We did not conduct a critical appraisal of study quality. However, the aim of the study was to gain a broad overview of the research available, rather than assess the quality. Finally, this review focused primarily on research relating to oral PrEP. Literature on the use of microbicides was not explored and may have produced different evidence on awareness, willingness and acceptability with different populations.

The sheer lack of TasP acceptability research demonstrates that it has been framed primarily as a clinical and health service delivery issue and tacitly assumes that people living with HIV will accept early treatment as an inevitability. Chen [26] reviewed research that explored the impact of treatment-related optimistic beliefs and risk of HIV transmission. However, this review focuses largely on the understandings of science and potential for risk compensation, rather than on the acceptability of using ARVs as a clinically-endorsed HIV prevention intervention at the point of diagnosis. Therefore, how people living with HIV might negotiate both their epidemiological and social role in HIV prevention, what additional burdens this might place on their physical and mental health, and how TasP as an intervention may play a role in reinforcing or refocusing social stigma around HIV onto the positive body is largely absent from the current scientific evidence.

The majority of PrEP research within trials focused on heterosexual women in serodiscordant couples, while most stand-alone studies concentrated almost exclusively on MSM. There were only two studies which included heterosexual men. It is noteworthy that willingness to use PrEP was reported to be high among MSM, suggesting it could be a popular HIV prevention method within this population. However, it is unclear why this may be the case. Knowledge and willingness in some studies were associated with education and age, but there were no overall consistent findings across studies. Age may still be an important factor to explore, although it is unclear whether there may be a generational effect. Future research should consider how knowledge of and/or previous experiences with HIV, particularly in relation to PEP [55], could influence PrEP use. Most studies were conducted in the US, and there was only one published articled on PrEP acceptability in the UK with any population at the time of the review. While there is research underway to address the imbalance, this has implications for considering where, and for whom, ARVs might offer a potential HIV prevention option.

This review found highly varied measures of acceptability, which makes it difficult to compare results across studies. This variation is supported by findings from Mensch et al. [35] who highlight that there is little consensus on the definition and operationalisation of acceptability for PrEP. However, different approaches to acceptability did not appear to affect the overall results in this review: the majority of studies reported high rates of willingness to use PrEP, regardless of whether participants were presented with a single willingness question, multiple questions or a range of PrEP scenarios. This suggests the need to further consider who is being asked about acceptability, how they respond to these questions and what motivates these responses. For instance, motivation to take or maintain adherence to PrEP may be affected by sero-discordant relationships [39] or engagement in high-risk sexual activity [48]. Questions about acceptability therefore need to push beyond simple willingness, to understand the context within which interventions such as PrEP might be acceptable and/or preferred. Only five studies provided specific information, including efficacy rates, for PrEP, whereas most did not specify these rates or did not report this. Two articles described how this information, and the way interventions are framed, will have significant implications for the acceptability and uptake of these interventions [53, 66]. Transparency and consistency in terms of how PrEP and TasP are presented to participants is therefore critical to understanding if, and in what circumstances, these interventions will be acceptable to potential users, and how they might or might not be used in combination with other prevention options. This will also require consideration of the environment in which PrEP and/or TasP are offered to potential users, including the acceptability of these interventions to health practitioners [71].

Acceptability was generally approached and assessed by considering: willingness to take PrEP or TasP; adherence to PrEP (or TasP); and subsequent or hypothetical risk compensation and/or disinhibition. These approaches focus on the ways in which the individual chooses and/or manages the use of the intervention and their sexual behaviour (hence their description as bio-behavioural) [32]. Although important, willingness or adherence alone will not necessarily translate into sustained and effective use of interventions; it is also critical to consider how individuals’ choices and actions are situated, constrained and/or enabled by the broader social context [4]. There was limited evidence of this in our review. Kippax and Stephenson [5] have argued not only that the broader social context needs to be considered, but that using ARVs for HIV prevention will necessarily require understanding and responding to the *social* context within which they might be used, describing the need for a *social public health* approach. In other words, the way in which individuals view, choose, and maintain the use of particular HIV prevention methods are significantly influenced by social, cultural and structural factors. Moreover, the social context within which negotiations of safer sex take place will also have a real impact on how interventions are sustained, and how sexual behaviour may be modified in light of these new prevention methods. This means that measurements of acceptability will need to move beyond expressions of willingness or adherence to medication, and consider the broader social, cultural and structural factors that will impact and shape the potential uptake and sustained effectiveness of PrEP and/or TasP. While the evidence is limited in this review, key factors include but are not limited to: social stigma, social pressures regarding sexual relationships, mistrust of medical settings and structural constraints including financial barriers and access.

Perceptions of risk and choice of risk management strategy appear to be key factors in adherence to PrEP [72]. However, risk perception and existing risk management by participants were discussed in a minority of articles in our review. As highlighted by Golub et al. [33] behavioural disinhibition and risk compensation present important differences in perceptions of risk and reasons for the choice and/or apparent absence of particular HIV risk management strategies. More nuanced understandings of risk management, including how multiple and conflicting risks might be managed, are therefore needed to better understand in what circumstances PrEP may be an appropriate intervention. In a community report which explored the experiences of black Africans in serodiscordant relationships living in the UK, Bourne et al. [73] described HIV positive-women’s anxieties that TasP might reduce their ability to negotiate condom use with discordant, male partners. These women were reluctant to rely on TasP as a prevention option, thus demonstrating how gender dynamics in serodiscordant relationships can play an important role in risk management strategies for HIV-positive women. These issues highlight how complicated social negotiations are embedded in starting and maintaining adherence to ARV prevention methods. Future acceptability research needs to consider the way in which diverse social contexts will play a significant role in the success of PrEP or TasP.

There was also limited evidence of the role structural factors may play in the acceptability of biomedical prevention. The studies that examined this suggested future trial design and decisions about the introduction of biomedical prevention into sexual health services need to be realistic about where adherence to PrEP is possible for individuals and where more structural approaches to HIV prevention may need to be pursued [38]. The need to identify the causes of poor adherence was stressed. If social structures form the biggest barriers to adherence, individual counselling and support will be unable to improve adherence rates. In addition, barriers such as access to health services or the cost of interventions were identified as playing an important role in the uptake of interventions. However, the variation across studies in terms of national and local health systems, and the extent to which cost was identified as a barrier, makes it difficult to offer general conclusions. This suggests the need to consider social and structural elements specific to national and local contexts. These findings echo Roberts and Matthews [74] who argue for the importance of structural HIV prevention interventions alongside biomedical and behavioural interventions.

## Conclusions

Literature on the acceptability of ARV prevention interventions amongst potential users focused largely on MSM (and to a lesser extent, heterosexual women in Africa), and was strikingly limited when it came to TasP. In order to strengthen understandings of how PrEP and/or TasP might be introduced and maintained as sustainable and effective HIV prevention interventions, further research is needed in five areas: engagement with people living with HIV in relation to potential TasP acceptability as well as perceptions of existing experiences with ARVs; further operationalisation of measures of acceptability to enable comparison between future studies and which move beyond measurements of adherence alone; further examination of what might motivate individuals to take PrEP, moving beyond categories such as age and education; the role of existing, complex (behavioural) risk management strategies in the adoption of biomedical prevention; and the potential role of social and structural influences above and beyond individual choices and/or behaviours, which would include examining the role of health providers in PrEP uptake and support. These five broad areas of research would help to further a social public health approach to acceptability and enable the identification of when, where and for whom ARVs for HIV prevention would be appropriate and effective. Our review suggests that, despite the issuing of guidelines for PrEP use in the US and the roll out of TasP at the clinical (if not the population) level, numerous questions remain as to just how acceptable, and therefore successful, these interventions will be in altering the course of the HIV epidemic. We have a responsibility to ensure that they are answered to achieve the true potential of biomedical HIV prevention.
